# Worse health status, sleeping problems, and anxiety in 16-year-old students are associated with chronic musculoskeletal pain at three-year follow-up

**DOI:** 10.1186/s12889-019-7955-y

**Published:** 2019-11-27

**Authors:** Julia S. Malmborg, Ann Bremander, M. Charlotte Olsson, Anna-Carin Bergman, A. Sofia Brorsson, Stefan Bergman

**Affiliations:** 1 The Rydberg Laboratory for Applied Sciences, Halmstad University, Box 823, SE-301 18 Halmstad, Sweden; 2 Spenshult Research and Development Center, Bäckagårdsvägen 47, SE-302 74 Halmstad, Sweden; 3 Department of Regional Health Research, University of Southern Denmark, Winsløvsparken 19.3, DK-5000 Odense, Denmark; 4 Danish Hospital for Rheumatic Diseases, University Hospital of Southern Denmark, Engelshøjgade 9A, DK-6400 Sønderborg, Denmark; 5 Department of Clinical Sciences Lund, Section of Rheumatology, Lund University, Box 117, SE-221 00 Lund, Sweden; 6 Primary Health Care Unit, Department of Public Health and Community Medicine, Institute of Medicine, The Sahlgrenska Academy, University of Gothenburg, Box 454, SE-405 30 Gothenburg, Sweden

**Keywords:** Chronic musculoskeletal pain, Health status, Sleep, Anxiety, Adolescent, Student, Epidemiology

## Abstract

**Background:**

Chronic musculoskeletal pain is common in adolescents, and it has been shown that adolescents with pain may become young adults with pain. Pain often coincides with psychosomatic symptoms in adults, but little is known about longitudinal associations and predictors of pain in adolescents. The aim was to investigate chronic musculoskeletal pain and its associations with health status, sleeping problems, stress, anxiety, depression, and physical activity in 16-year-old students at baseline, and to identify risk factors using a three-year follow-up.

**Methods:**

This was a longitudinal study of 256 students attending a Swedish upper secondary school. Questionnaires regarding chronic musculoskeletal pain and distribution of pain (mannequin), health status (EQ-5D-3 L), sleeping problems (Uppsala Sleep Inventory), stress symptoms (single-item question), anxiety and depression (Hospital Anxiety and Depression Scale), and physical activity (International Physical Activity Questionnaire) were issued at baseline and follow-up. Student’s t-test and chi^2^ test were used for descriptive statistics and logistic regression analyses were used to study associations between chronic pain and independent variables.

**Results:**

Fifty-two out of 221 students at baseline (23.5%) and 39 out of 154 students at follow-up (25.3%) were categorized as having chronic musculoskeletal pain. Chronic musculoskeletal pain at follow-up was separately associated with reporting of an EQ-5D value below median (OR 4.06, 95% CI 1.83–9.01), severe sleeping problems (OR 3.63, 95% CI 1.69–7.82), and possible anxiety (OR 4.19, 95% CI 1.74–10.11) or probable anxiety (OR 3.82, 95% CI 1.17–12.48) at baseline. Similar results were found for associations between chronic musculoskeletal pain and independent variables at baseline. In multiple logistic regression analysis, chronic musculoskeletal pain at baseline was a predictor of chronic musculoskeletal pain at follow-up (OR 2.99, 95% CI 1.09–8.24, R^2^ = 0.240).

**Conclusion:**

Chronic musculoskeletal pain at baseline was the most important predictor for reporting chronic musculoskeletal pain at the three-year follow-up, but a worse health status, severe sleeping problems, and anxiety also predicted persistence or development of chronic musculoskeletal pain over time. Interventions should be introduced early on by the school health services to promote student health.

## Background

Since the mid-eighties, there has been a doubling of reports of physical and psychosomatic problems such as pain, sleep disturbances, stress, and depression in young adolescents in Sweden. Girls appear to be more affected, but such problems have also increased in boys [1] . There is a need to investigate whether these trends continue in Swedish upper secondary school students (16 to 19-year-olds) who are enrolled in university preparatory programs and vocational programs.

The prevalence of chronic musculoskeletal pain (CMP) in children and adolescents ranges from 4 to 40% [2]. It is important to treat pain at the early ages to prevent it from persisting into adulthood [3]. Pain is a bio-psychosocial state that may co-exist with sleeping problems [4, 5], stress, anxiety, depression [6] and/or a worse health status [7] in adults, but less is known about the long-term effects and about factors that may be predictors of pain development in adolescents.

Results in recent years have shown that fifteen-year-old adolescents go to bed later, do not get the recommended amount of sleep, and have more difficulty in falling asleep than their counterparts in the eighties [8]. Recommendations state that adolescents should sleep for 8–10 h to retain optimal levels of learning, attention, and good health [9]. Adolescents with chronic pain experience more sleeping problems than adolescents with no pain [10], and the number of regions with pain and the intensity of pain experienced both contribute to poorer sleep [11]. The relationship between pain and sleep appears to be bidirectional, and there has been a lack of studies investigating sleeping problems as a predictor of development of CMP in adolescents [12].

Psychosomatic problems such as stress, anxiety, and depression are common in adolescents and they appear to be reported by girls more frequently than by boys [13]. Adolescents with musculoskeletal pain have been found to experience higher levels of stress than adolescents with no pain [14], and pain has been found to be moderately correlated with anxiety [13] in cross-sectional studies. Moreover, adolescents with symptoms of anxiety and depression are more likely to experience CMP in multiple sites on the body [15], but the mechanisms behind these associations still remain to be explained.

High levels of physical activity and sports participation are associated with a higher degree of well-being in European adolescents [16]. However, in the same study 86% of adolescents did not reach recommended levels of physical activity, and girls were less active than boys [16]. Even though physical activity is generally a predictor of health, musculoskeletal pain may have a negative effect on this relationship. Adolescent athletes who frequently experienced pain reported having a worse health status than adolescent athletes with no pain [17], highlighting the need for assessment of these factors together.

Physical and psychosomatic problems are common in adolescents, but more research is needed to investigate longitudinal relationships. The aim of this study was to investigate chronic musculoskeletal pain and its associations with health status, sleeping problems, stress, anxiety, depression, and physical activity in 16-year-old students at baseline and to identify risk factors at baseline that were associated with persistence or development of chronic musculoskeletal pain at follow-up.

## Methods

### Study population

All the students who attended their first of 3 years at a Swedish upper secondary school (16 years old; *n* = 296) in 2011 were asked to participate in this longitudinal cohort study. The study took place in the south-west of Sweden. Students were enrolled in either university preparatory programs or vocational programs. Participation in the study was voluntarily, and all the students who agreed to participate signed a written informed consent document in 2013. The informed consent document stated that results from questionnaires used in quality work at the school in 2011 could be included in the study. The research project was approved by the Regional Ethical Review Board in Lund, Sweden (Dnr 2013/34), and it was carried out in accordance with the ethical guidelines of the World Medical Association (Declaration of Helsinki). Questionnaires were collected in the autumn of 2011 (baseline) and in the spring of 2014 (follow-up). The questionnaires were distributed digitally, and each student was given a unique login code.

### Questionnaires

CMP, health status, sleep, stress, anxiety and depression, and physical activity were all assessed through questionnaires.

CMP was evaluated using two validated questions. The first question was: “Have you experienced pain lasting more than 3 months during the last 12 months?”. The response options were “yes”, “no”, and “do not know”. The second question included a mannequin with 18 predefined regions and was: “Mark all the places on your body where you have experienced pain for more than 3 months in the last 12 months” [18].

The EQ-5D-3 L (EQ-5D) questionnaire assesses health status and covers mobility, self-care, usual activities, pain/discomfort, and anxiety/depression. In each of the five domains, no problems, some problems, or extreme problems are indicated by the participant, and the questions refer to the current state of health. Evaluation is done using an index ranging from 0 to 1.00, where 1.00 is complete health [19]. The minimum relevant difference for the index is 0.074, ranging from − 0.011 to 0.140 [20]. A numerical rating scale (NRS-11, with 0 representing the worst imaginable health state and 100 representing the best imaginable health state) was also used to measure health status.

Sleeping problems were assessed using questions from the Uppsala Sleep Inventory. The level of severity of sleeping problems (with a score of 1 = no problems, 2 = minor problems, 3 = moderate problems, 4 = severe problems, and 5 = very severe problems) regarding (a) initiating sleep, (b) maintaining sleep, (c) early awakening, and (d) restoration after sleep was assessed in the questionnaire [21].

Experiencing of stress was measured with the following single-item question: “Stress means a situation in which a person feels tense, restless, nervous or anxious or is unable to sleep at night because his/her mind is troubled all the time. Do you feel this kind of stress these days?”. The five response options ranged from “not at all” to “very much” [22].

The Hospital Anxiety and Depression Scale (HADS) is a 14-item questionnaire that provides one scale for anxiety and one for depression. Each item has four response options graded from 0 to 3, and each scale is scored between 0 and 21 points. Regarding cut-off values, 0–7 points are considered to indicate normal health in this respect (non-cases), 8–10 points are considered to indicate a possible mood disorder (possible cases), and ≥ 11 points are considered to indicate a probable state of anxiety or depression (probable cases) [23]. It is emphasized that the instrument should be used as a screening tool and not as a diagnostic tool [24]. The questionnaire has been validated in adolescents in a UK setting [25].

The International Physical Activity Questionnaire (IPAQ) short form [26] assesses time spent in walking, moderate physical activity (MPA), and vigorous physical activity (VPA) in the last week. Reported time was converted into metabolic equivalent of task (MET) by multiplication of duration and frequency of walking, MPA, and VPA (MET-minutes per week). Total physical activity (total PA) was equal to the sum of MET-minutes per week for walking, MPA, and VPA. Physical activity was also categorized as being low, moderate, or high category based on the IPAQ guidelines. Sitting time in the last week was also assessed [26].

### Statistical analysis

**Fig. 1:**
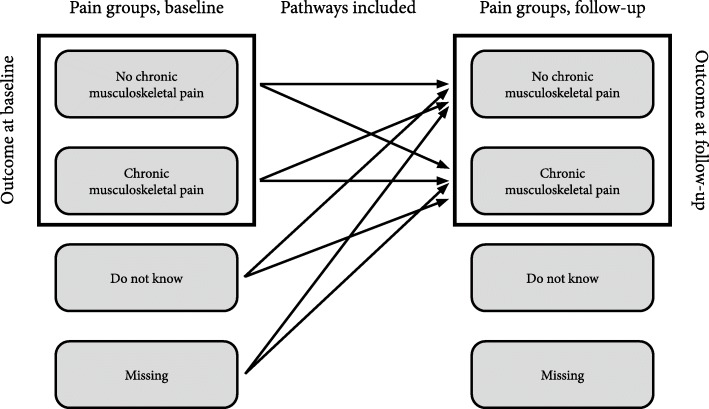
Description of pain groups included in analyses (black squares) and the pathways included in the outcome of pain groups at follow-up (black arrows)

At baseline and follow-up, students were grouped as having CMP (pain for more than 3 months in the previous 12 months) or not having CMP in the previous 12 months. Students who answered “do not know” regarding CMP at baseline or who were missing were excluded from cross-sectional pain analyses, but they were included in longitudinal analyses if they had answered “no” or “yes” regarding the CMP question at follow-up (Fig. 1).

EQ-5D was dichotomized into above and below the median. Stress and sleeping items were dichotomized into 1–3 points (best) vs. 4–5 points (worst). Individuals who scored as having at least severe problems (4 points) for one or more sleeping items were classified as having severe sleeping problems. HADS was categorized as non-cases, possible, and probable cases for anxiety and depression respectively.

The Shapiro-Wilks test revealed that the baseline data were not normally distributed, but due to having groups of more than 30 individuals, results for descriptive comparisons between boys and girls were expressed as mean ± standard deviation (SD) and analyzed with Student’s t-test. Chi^2^ tests were used to analyze differences in distribution between groups. Logistic regression analysis (adjusted for sex) with CMP as dependent variable and separately inserted independent variables was used, and also multiple logistic regression analysis with 95% confidence intervals (CIs). Pearson’s phi-coefficient was used to investigate intercollinarity between variables. Statistical analyses were performed with IBM SPSS Statistics v.24.0 (IBM Corp., Armonk, NY, USA) and statistical significance was assumed at *p*-values of < 0.05. Post-hoc power analysis revealed a statistical power of > 80% for baseline analyses assuming differences of at least one-half SD.

## Results

**Fig. 2:**
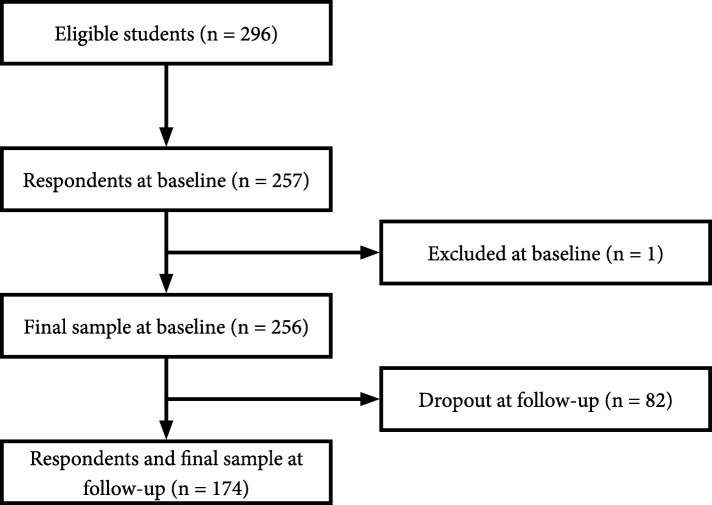
Flow chart of participants

Two hundred and fifty-seven of 296 students who were eligible participated in the study. One student was excluded as an outlier, leaving 256 students (87 boys and 169 girls; mean age 16.1 years, SD 0.6, range 15–20 years) at baseline. One hundred and seventy-four students (56 boys and 118 girls) participated in the study at follow-up (with a 32.0% dropout rate; Fig. 2).

### Differences between boys and girls

Descriptive data and comparisons between boys and girls at baseline and follow-up are listed in Tables 1 and 2. The self-rated health status of girls was worse than that of boys, both at baseline and at follow-up. In addition, at baseline the girls found it more difficult to initiate sleep than the boys (Table 1). The VPA and total PA reported by boys were higher than those reported by girls (Table 1), and boys were more frequently categorized as having a high level of physical activity at baseline and at follow-up (Table 2). Half of the girls were classified as being either a possible case or a probable case of anxiety at baseline, something that was not seen to the same extent in boys (Table 2).

**Table 1:** Results for health status, sleep, stress, HADS, and IPAQ for all students and for boys and girls separately at baseline and at follow-up. Results were analyzed with Student’s t-test and are presented as mean ± SD

	Baseline				Follow-up			
	All (*n* = 256) Mean ± SD	Boys (*n* = 87) Mean ± SD	Girls (*n* = 169) Mean ± SD	Boys vs. girls *p*-value	All (*n* = 174) Mean ± SD	Boys (*n* = 56) Mean ± SD	Girls (*n* = 118) Mean ± SD	Boys vs. girls *p*-value
Health status^a^								
EQ-5D (0.00–1.00)	0.84 ± 0.18	0.87 ± 0.17	0.83 ± 0.18	0.051	0.84 ± 0.20	0.89 ± 0.20	0.81 ± 0.19	0.022
NRS-11 (0–100)	78.1 ± 17.4	82.6 ± 15.4	75.8 ± 17.9	0.003	78.0 ± 17.6	82.7 ± 17.6	75.8 ± 17.3	0.015
Sleeping problems^b^ (1–5)								
Initiating sleep	2.2 ± 1.1	1.9 ± 1.0	2.4 ± 1.0	< 0.001	2.1 ± 1.0	2.0 ± 1.1	2.2 ± 1.0	0.249
Maintaining sleep	1.7 ± 1.0	1.4 ± 0.8	1.8 ± 1.0	0.007	1.9 ± 1.1	1.7 ± 1.0	2.0 ± 1.2	0.088
Early awakening	1.6 ± 1.0	1.6 ± 1.0	1.7 ± 1.0	0.843	1.7 ± 1.0	1.6 ± 1.0	1.8 ± 1.1	0.400
Non-restorative sleep	2.7 ± 1.2	2.6 ± 1.3	2.7 ± 1.2	0.704	2.8 ± 1.2	2.9 ± 1.2	2.8 ± 1.1	0.622
Stress^b^ (1–5)	2.5 ± 1.3	2.2 ± 1.2	2.7 ± 1.2	0.007	2.7 ± 1.2	2.4 ± 1.2	2.8 ± 1.2	0.072
HADS^b^ (0–21)								
Anxiety	6.8 ± 3.5	5.8 ± 3.0	7.3 ± 3.7	< 0.001	7.1 ± 3.8	6.4 ± 4.2	7.5 ± 3.6	0.075
Depression	3.7 ± 2.7	3.5 ± 2.6	3.8 ± 2.8	0.489	3.6 ± 3.1	3.7 ± 2.8	3.6 ± 3.2	0.877
IPAQ^c^								
Walking	553 ± 624	459 ± 558	602 ± 652	0.083	611 ± 622	607 ± 635	612 ± 619	0.964
MPA	547 ± 622	573 ± 618	534 ± 625	0.634	614 ± 634	824 ± 763	516 ± 540	0.009
VPA	1689 ± 1468	2451 ± 1579	1292 ± 1237	< 0.001	1784 ± 1496	2751 ± 1599	1334 ± 1210	< 0.001
Total PA	2790 ± 2111	3483 ± 2117	2428 ± 2021	< 0.001	3009 ± 2134	4182 ± 2218	2462 ± 1864	< 0.001
Sitting minutes per day	414 ± 221	426 ± 226	409 ± 219	0.562	522 ± 218	491 ± 216	536 ± 218	0.201

^a^ Scored from worst to best

^b^ Scored from best to worst

^c^ Walking, moderate physical activity (MPA), vigorous physical activity (VPA), and total physical activity (Total PA) as metabolic equivalent of task (MET) minutes per week

**Table 2:** Results for categories in HADS anxiety, HADS depression, IPAQ, and CMP for boys and girls at baseline and at follow-up. Results were analyzed with Chi^2^ test and are presented as n (%)

	Baseline			Follow-up		
	Boys n (%)	Girls n (%)	Boys vs. girls *p*-value	Boys n (%)	Girls n (%)	Boys vs. girls *p*-value
HADS anxiety	**(*n*** **= 87)**	**(*n*** **= 169)**		**(*n*** **= 56)**	**(*n*** **= 118)**	
Non-cases	73 (83.9%)	94 (55.6%)		35 (62.5%)	61 (51.7%)	
Possible cases	8 (9.2%)	47 (27.8%)	< 0.001	12 (21.4%)	38 (32.2%)	0.311
Probable cases	6 (6.9%)	28 (16.6%)		9 (16.1%)	19 (16.1%)	
HADS depression	**(*n*** **= 87)**	**(*n*** **= 169)**		**(*n*** **= 55)**	**(*n*** **= 118)**	
Non-cases	79 (90.8%)	148 (87.6%)		49 (89.1%)	107 (90.7%)	
Possible cases	7 (8.0%)	18 (10.7%)	^a^	3 (5.5%)	7 (5.9%)	^a^
Probable cases	1 (1.1%)	3 (1.8%)		3 (5.5%)	4 (3.4%)	
IPAQ	**(*n*** **= 87)**	**(*n*** **= 167)**		**(*n*** **= 55)**	**(*n*** **= 118)**	
Low	6 (6.9%)	31 (18.6%)		3 (5.5%)	20 (16.9%)	
Moderate	14 (16.1%)	54 (32.3%)	< 0.001	5 (9.1%)	43 (36.4%)	< 0.001
High	67 (77.0%)	82 (49.1%)		47 (85.5%)	55 (46.6%)	
CMP	**(*n*** **= 79)**	**(*n*** **= 142)**		**(*n*** **= 52)**	**(*n*** **= 102)**	
No	64 (81.0%)	150 (73.9%)	0.235	42 (80.8%)	73 (71.6%)	0.214
Yes	15 (19.0%)	37 (26.1%)		10 (19.2%)	29 (28.4%)	

^a^For HADS depression, it was not possible to use the Chi^2^ test due to the small groups

### Chronic musculoskeletal pain

Two hundred and twenty-one students of the 256 enrolled could be categorized as either having CMP or not having CMP at baseline (12.9% answered “do not know” and 0.8% had missing answers), and 154 out of 174 at follow-up (10.9% answered “do not know” and 0.6% had missing answers). At baseline, 169 (76.5%) were placed in the no-CMP group and 52 (23.5%) were placed in the CMP group, and at follow-up 115 (74.7%) were placed in the no-CMP group and 39 (25.3%) in the CMP group. There was no significant difference between the distribution of boys and girls reporting having CMP at baseline or at follow-up (Table 2). Of those who reported having CMP at baseline, 13 (25%) reported having one pain region and 39 (75%) reported having two or more pain regions. At follow-up, 14 (36%) reported having one pain region and 25 (64%) reported having two or more pain regions. The most common pain regions at baseline for boys were the knees (7.6%), lower legs/feet (7.6%), anterior chest (5.1%), and shoulders/upper arms (5.1%), and for girls they were the neck (11.3%), knees (10.6%), and lower back (9.9%).

### Transitions of chronic musculoskeletal pain over 3 years

Both persistence of CMP and its development over time were analyzed in the follow-up. There were therefore four major transition pathways included between baseline and follow-up: (1) “no CMP”/“do not know”/missing at baseline to “no CMP” at follow-up (*n* = 103, 66.9%), (2) “no CMP”/“do not know”/missing at baseline to “CMP” at follow-up (*n* = 24, 15.6%), (3) “CMP” at baseline to “CMP” at follow-up (*n* = 15, 9.7%), and (4) “CMP” at baseline to “no CMP” at follow-up (*n* = 12, 7.8%).

### Associations with and predictors of chronic musculoskeletal pain

CMP at baseline and associations with health status, sleep, stress, anxiety, depression, and physical activity were analyzed with logistic regression analysis, controlling for sex and with independent variables inserted separately in the analysis. CMP was separately associated with reporting an EQ-5D value below the median, severe sleeping problems, high level of stress, possible anxiety, probable anxiety, and possible depression at baseline. When analyzing the results using multiple logistic regression, CMP was still significantly associated with an EQ-5D value below the median and with belonging to the groups of possible or probable cases of anxiety (Table 3).

**Table 3:** Associations between background variables at baseline and CMP at baseline (*n* = 221) based on logistic regression analysis

Baseline variables	Model 1 No CMP at baseline = 0 (*n* = 169) CMP at baseline = 1 (*n* = 52)		Model 2 No CMP at baseline = 0 (*n* = 169) CMP at baseline = 1 (*n* = 52)	
	OR	(95% CI; *p*-value)	OR	(95% CI; *p*-value)
Sex				
Boys	1.00		1.00	
Girls	1.50	(0.77–2.95; *p* = 0.237)	0.86	(0.39–1.89; *p* = 0.71)
EQ-5D				
EQ-5D ≥ 0.85	1.00		1.00	
EQ-5D < 0.85	5.04	(2.59–9.80; *p* < 0.001)	3.61	(1.76–7.41; *p* < 0.001)
Problems initiating sleep				
None to moderate	1.00			
Severe to very severe	2.51	(1.09–5.77; *p* = 0.030)		
Problems maintaining sleep				
None to moderate	1.00			
Severe to very severe	1.79	(0.57–5.65; *p* = 0.318)		
Problems with early morning awakening				
None to moderate	1.00			
Severe to very severe	2.37	(0.85–6.61; *p* = 0.099)		
Problems with non-restorative sleep				
None to moderate	1.00			
Severe to very severe	3.69	(1.91–7.13; *p* < 0.001)		
Severe sleeping problems				
No severe sleeping problems	1.00		1.00	
Severe sleeping problems	3.06	(1.61–5.83; *p* = 0.001)	1.81	(0.86–3.81; *p* = 0.121)
Stress				
Points 1–3	1.00		1.00	
Points 4–5	2.36	(1.17–4.75; *p* = 0.016)	0.79	(0.31–2.01; *p* = 0.617)
HADS anxiety				
Non-cases	1.00		1.00	
Possible cases	3.42	(1.55–7.57; *p* = 0.002)	2.60	(1.08–6.30; *p* = 0.034)
Probable cases	6.49	(2.63–16.02; *p* < 0.001)	3.32	(1.02–10.84; *p* = 0.047)
HADS depression				
Non-cases	1.00			
Possible cases	2.76	(1.09–7.01; *p* = 0.033)		
Probable cases	1.83	(0.16–20.90; *p* = 0.625)		
IPAQ				
Low	1.00			
Moderate	0.79	(0.27–2.33; *p* = 0.668)		
High	1.60	(0.62–4.09; *p* = 0.331)		

Model 1: A logistic regression analysis in which variables were controlled for sex but otherwise included separately in the analysis

Model 2: A multiple logistic regression analysis. R^2^ (Nagelkerke) = 0.233 for full model

*OR* odds ratio, *CI* confidence interval

The persistence or development of CMP in the follow-up, controlled for sex, was separately predicted by having an EQ-5D value below the median, severe sleeping problems, possible anxiety, probable anxiety, and CMP at baseline. When performing a multiple logistic regression analysis with variables controlled for each other and for sex, CMP at baseline remained as a significant predictor of persistence or development of CMP at follow-up (Table 4).

**Table 4:** Associations between background variables at baseline and CMP at follow-up (*n* = 153–154) based on logistic regression analysis

Baseline variables	Model 1 No CMP at follow-up = 0 (*n* = 115) CMP at follow-up = 1 (*n* = 39)		Model 2 No CMP at follow-up = 0 (*n* = 115) CMP at follow-up = 1 (*n* = 38)	
	OR	(95% CI; *p*-value)	OR	(95% CI; *p*-value)
Sex				
Boys	1.00		1.00	
Girls	1.67	(0.74–3.76; *p* = 0.217)	1.05	(0.41–2.67; *p* = 0.916)
EQ-5D				
EQ-5D ≥ 0.85	1.00		1.00	
EQ-5D < 0.85	4.06	(1.83–9.01; *p* = 0.001)	2.04	(0.77–5.36; *p* = 0.149)
Problems initiating sleep				
None to moderate	1.00			
Severe to very severe	3.44	(1.24–9.54; *p* = 0.018)		
Problems maintaining sleep				
None to moderate	1.00			
Severe to very severe	3.04	(0.41–22.62; *p* = 0.277)		
Problems with early morning awakening				
None to moderate	1.00			
Severe to very severe	2.69	(0.77–9.44; *p* = 0.122)		
Problems with non-restorative sleep				
None to moderate	1.00			
Severe to very severe	2.60	(1.18–5.70; *p* = 0.018)		
Severe sleeping problems				
No severe sleeping problems	1.00		1.00	
Severe sleeping problems	3.63	(1.69–7.82; *p* = 0.001)	2.06	(0.84–5.07; *p* = 0.115)
Stress				
Points 1–3	1.00			
Points 4–5	1.75	(0.76–4.02; *p* = 0.187)		
HADS anxiety				
Non-cases	1.00		1.00	
Possible cases	4.19	(1.74–10.11; *p* = 0.001)	2.31	(0.86–6.20; *p* = 0.095)
Probable cases	3.82	(1.17–12.48; *p* = 0.027)	1.27	(0.31–5.24; *p* = 0.740)
HADS depression				
Non-cases	1.00			
Possible cases	1.61	(0.45–5.72; *p* = 0.465)		
Probable cases	–			
IPAQ				
Low	1.00			
Moderate	2.39	(0.60–9.58; *p* = 0.217)		
High	1.64	(0.42–6.41; *p* = 0.476)		
CMP				
No CMP	1.00		1.00	
CMP	5.51	(2.23–13.61; *p* < 0.001)	2.99	(1.09–8.24; *p* = 0.034)
Do not know or missing	1.45	(0.42–4.99; *p* = 0.556)	0.79	(0.18–3.49; *p* = 0.756)

Model 1: A logistic regression analysis in which variables were controlled for sex but otherwise included separately in the analysis

Model 2: A multiple logistic regression analysis. R^2^ (Nagelkerke) = 0.240 for full model

*OR* odds ratio, *CI* confidence interval

## Discussion

In this longitudinal study of 16-year-old Swedish upper secondary school students, the prevalence of CMP at baseline and at the three-year follow-up was about 25% and there was no significant difference in prevalence between boys and girls. CMP at baseline was associated with reporting a worse health status, severe sleeping problems, a high level of stress, anxiety, and possible depression. A worse health status and anxiety stood out as important factors. From a three-year perspective, persistence or development of pain was predicted by a worse health status, severe sleeping problems, anxiety, and CMP at baseline. Of these factors, CMP at baseline appeared to be the most important predictor.

The prevalence of CMP in this study is in line with previous findings. King et al. [2] reported that the prevalence of back pain ranged between 14 to 24% and musculoskeletal/limb pain from 4 to 40%, that pain increased with age, and that it was more prevalent in girls. The prevalence of chronic pain reported by Østerås et al. [14] was 36%, whereas Harrison, Wilson and Munafo [27] reported a prevalence of CMP of 6% for study populations of similar ages to those in the present study. In the previous literature, the results have been inconclusive and have varied with the type of population and methodology used, but the prevalence found in our study appears to be in the middle of the ranges reported earlier. Interestingly, there were no significant differences between prevalence of CMP in boys and girls in the present study, despite there being an overrepresentation of girls. This is not in line with previous findings, where girls report pain more often than boys [2, 28].

Pain co-varies with a worse health status in adolescents [17, 29] and adults [7]. In the present study, worse health status was associated with reporting of CMP at baseline, even when we controlled for sleep, stress, and anxiety. Population data for EQ-5D for Swedish adolescents are based on the youth version [30]. When this study was set up, the youth version was not completely implemented in Sweden, so the adult version of EQ-5D was used. The two versions are similar, but no comparisons with population norms for Swedish adolescents can be done. The adult version has been used in a previous study involving 16-year-olds [29].

Sleeping problems (controlled for sex) increased the risk of reporting CMP over 3 years. Due to the prevalence of CMP in the present study, we analyzed both persistence and development. Because of this, we do not know if sleeping problems without CMP at baseline is predictive of CMP at follow-up. In a systematic review, Andreucci et al. [12] reported that having sleeping problems did not predict onset of musculoskeletal pain in adolescents, but that assessment methods were not as developed as in studies on adults. Sleeping problems appear to be associated with persistence of pain in children and adolescents [10], and more studies on this subject are needed.

Stress was not found to be a predictor over time of CMP in this study, but (controlled for sex) it was associated with CMP at baseline, a finding that has also been described by Østerås et al. [14]. Stress arising from school-related demands was found to be common in older adolescents enrolled in academic or vocational programs, but there was only a moderate correlation between stress and musculoskeletal pain [13]. Peer-related stress mediated by worry has been found to be predictive of musculoskeletal pain in younger adolescents [31], but there is a need to investigate longitudinal associations further.

Thirty-five per cent of the students reported having symptoms of anxiety and 11% depression (possible or probable) at baseline. This is higher than the previously reported prevalence of 6% for anxiety, but similar to the prevalence of 10% for depression in European adolescents [32]. However, Wiklund et al. [13] reported a higher prevalence of anxiety in adolescents than in the present study. Symptoms of anxiety and depression co-vary with increased odds for reporting chronic multisite pain in adolescents [15], and in the present study we found similar results for CMP at baseline. Furthermore, anxiety at baseline was still significantly associated with CMP, even when accounting for other variables such as stress and health status. Auvinen et al. [33] found that multisite pain over time was a predictor of higher levels of anxiety, and they reported a prevalence of anxiety of 30%. They did not assess anxiety at baseline, and there may have been a bidirectional relationship between pain and anxiety.

The boys in our study were found to be more physically active than girls, which has also been shown in other studies as well [16]. Participation in sports or a high level of physical activity is often seen as a confounding factor for pain [34, 35], but the level of physical activity did not seem to impact on reporting of CMP in the present study. We did not ask about sports participation, and we do not know whether this would have contributed to a better understanding of the results regarding physical activity and pain.

### Strengths and limitations

The main strengths of the study were its longitudinal design, the high participation rate, and the low internal dropout rate. Eighty-seven per cent of the students who were eligible participated in the study at baseline, and 68% of the students for whom we had baseline data collected participated in the follow-up. The web-based questionnaire may have been one reason for the low internal dropout rate.

Another strength was that the school included in this study enrolls students from the whole community and from different housing areas. Even if not all seventeen national programs were represented, students from a variety of programs participated in the study. Because of this variation, we found it valid and interesting that one out of four students were found to have chronic musculoskeletal pain.

Due to the small numbers available within this sample, an even larger sample would have been needed for subgroup analysis of persistence and development of CMP. A larger sample could also have helped to achieve more power in multiple regression analyses. Number of pain sites was omitted from analyses because of the relatively low number of subjects with CMP. There were no significant differences in the prevalence of CMP at baseline between those who responded to the questionnaires and those who did not at follow-up. However, those who did not respond at follow-up reported having a worse health status at baseline, and this should be considered when interpreting the results.

There are some methodological concerns that should to be addressed. The HADS instrument is designed to be assessed as two scales, but it has been proposed to be a unidimensional measure of general distress [36, 37]. The instrument cannot be used as a diagnostic tool [24], and the results should be interpreted as being symptoms of anxiety and depression rather than as any medical diagnosis.

Sitting time from IPAQ only assesses the total time of sitting still rather than bouts of sitting, which is more interesting from a health perspective. Furthermore, the guidelines for IPAQ does not support dichotomizing the results from sitting time, therefore it was omitted from analysis.

Screen use before bedtime was not assessed in this study, which may be a limitation with regard to sleep assessment. The duration of sleep decreases in adolescents with increasing screen use, but there are still methodological issues that must be resolved in order to determine whether there are causal effects [38]. When this study was carried out in 2011 it was difficult to foresee how smartphones and tablets were going to change the way adolescents live their lives. In retrospect, it would have been interesting to include screen use in this study. Unfortunately it was not recognized as an interesting factor at that moment, which is a limitation.

## Conclusions

One in four 16-year-old students reported having CMP, and this was associated with having a worse health status, severe sleeping problems, and anxiety at baseline. CMP at baseline was the most important predictor for reporting CMP at the three-year follow-up, but a worse health status, severe sleeping problems, and anxiety also predicted persistence or development of CMP over time. Interventions should be introduced early on by the school health services to promote student health.

## Data Availability

The datasets generated and/or analyzed during the current study are not publicly available for ethical reasons.

## Abbreviations

CI: Confidence interval
CMP: Chronic musculoskeletal pain
EQ-5D: EQ-5D-3 L
HADS: Hospital Anxiety and Depression Scale
IPAQ: International Physical Activity Questionnaire
MET: Metabolic equivalent of task
MPA: Moderate physical activity
NRS: Numerical rating scale
OR: Odds ratio
SD: Standard deviation
Total PA: Total physical activity
VPA: Vigorous physical activity
